# The Role of Psychological Health in Cardiovascular Health: A Racial Comparison

**DOI:** 10.3390/healthcare13080846

**Published:** 2025-04-08

**Authors:** Briana N. Sprague, Kelly M. Mosesso

**Affiliations:** 1 Division of General Internal Medicine and Geriatrics, Department of Medicine, Indiana University School of Medicine, Indianapolis, IN 46202, USA; 2 Indiana University Center for Aging Research, Regenstrief Institute, Inc., Indianapolis, IN 46202, USA; 3 Department of Biostatistics, Indiana University School of Medicine, Indianapolis, IN 46202, USA

**Keywords:** cardiovascular health, life’s essential 8, positive psychological health, negative psychological health, middle age

## Abstract

**Purpose**: Modifiable health factors influence racial disparities in cardiovascular health (CVH), yet the role of psychological health in these disparities remains understudied. This study examines (1) the association between negative and positive psychological health measures and CVH and (2) the racial differences in these associations among US adults. **Methods**: Aim 1 included adults aged 34–84 from the MIDUS biomarker substudy (n = 1255). Aim 2 included adults aged 28–84 from the MIDUS parent study (N = 4702). Our outcome was CVH, operationalized as the AHA’s Life’s Essential 8 (LE8) total score, behavior, and health factor subscores. Negative psychological health was operationalized as depressive symptoms (CES-D), stress reactivity (from the Multidimensional Personality Questionnaire [MPS]), aggression (from the MPS), pessimism (Life Orientation Test), perceived stress (Perceived Stress Scale), and trait anxiety (Spielberger Trait Anxiety Inventory); positive psychological health was operationalized as psychological well-being (“PWB”; Ryff Well-Being Scale [WBS] and MPS), purpose in life (from the WBS), mindfulness (developed by MIDUS), gratitude (developed by MIDUS), and optimism (Life Orientation Test). **Results**: In covariate-adjusted models, most negative psychological health factors were negatively associated with LE8 total scores and health behavior subscores. Of those, pessimism was the only factor to demonstrate Black–White differences (Black > White, *p* < 0.001). Positive psychological health factors were less consistently associated with the LE8 total, health behavior, and health factor subscores in covariate-adjusted models. Of these, PWB (Black > White, *p* < 0.001), gratitude (Black > White, *p* < 0.001), and optimism (Black > White, *p* < 0.001) demonstrated significant differences by race. **Conclusions**: Black–White differences in LE8 are not largely explained by differences in psychological health.

## 1. Introduction

In 2022, the American Heart Association (AHA) updated its concept of cardiovascular health (CVH) to reflect eight health components: (1) diet, (2) physical activity, (3) nicotine exposure, (4) sleep health, (5) body mass index (BMI), (6) blood lipids, (7) blood glucose, and (8) blood pressure [1]. While poor CVH as assessed by “Life’s Essential 8” (LE8) is prevalent among American adults broadly, sociodemographic disparities are present, as Black adults have poorer CVH compared to White adults [2]. These disparities are largely replicated when considering specific health components, where Black adults generally report less adherence to a high-quality diet [3,4,5], less physical activity engagement [3,5], poorer sleep quality [6,7,8], greater obesity rates [9,10,11], elevated blood pressure [12,13], and elevated HbA1_c_ levels [14,15], compared to White adults. Although less consistent across the literature, there is also evidence of greater tobacco use [3,5,16,17] and dyslipidemia [18,19,20,21,22] among Black compared to White adults.

Since CVH fluctuates over time [23], disparities may be more pronounced at specific life stages. Aggregating data across adulthood could obscure critical periods when interventions are most effective. Midlife is one possible critical period, as this is a period where poor CVH is prevalent [1] and CVH loss may accelerate [24]. Individuals at midlife with poor CVH also experience greater lifetime risk of developing CVD compared to older adults with the same CVH profile [25,26], suggesting that midlife may reflect a critical period for understanding CVH indices that contribute to racial health disparities.

The association between negative psychological factors such as depression, anxiety, and perceived stress (and others) with CVH are well-established [27,28,29]. While systemic racism puts Black individuals at a higher risk of experiencing negative psychological health [30], not everyone goes on to develop poor CVH. Growing evidence suggests that positive psychological factors such as well-being, optimism, and vitality may be upstream determinants of CVH as they influence perceptions, interpersonal interactions, and the ability to participate in health behaviors [1,31,32,33]. For example, prospective cohort studies demonstrate that greater psychological well-being (e.g., optimism, purpose in life, and positive affect) is related to various cardiovascular health outcomes [34,35,36,37,38]. However, the link has not been well established, partly owing to both the heterogenous assessment of cardiovascular outcomes measures and the limited range of well-being measures [39,40]. A better understanding of which psychological health measures are associated with CVH can inform future intervention targets for CVH promotion [39]. Unlike prior studies that have examined psychological health and cardiovascular health separately, this study integrates both positive and negative psychological health constructs within the LE8 framework, providing a more comprehensive understanding of their role in racial health disparities. This work is novel in its examination of psychological health correlates across multiple domains and its evaluation of whether racial differences persist after covariate adjustment.

### Present Study

The first goal of this study was to identify which measures of psychological health were associated with CVH as assessed by one’s LE8 scores, both bivariately as well as covariate-adjusted (except race due to too few non-White participants). Then, using the parent MIDUS study, we evaluated whether there were significant racial differences in the psychological health correlates after covariate adjustment. Together, this will help identify psychological factors related to the various components of CVH and may also contribute to underlining racial CVH disparities.

## 2. Methods

### 2.1. Data and Analytic Sample

This study was conducted using secondary data from the Midlife in the United States (MIDUS) study, a national longitudinal study examining health and well-being. The first wave of data collection (MIDUS-1) occurred in 1995–1996 and included 7108 participants drawn from four subsamples as follows: (1) a national probability sample recruited via random-digit dialing (n = 3487), (2) oversamples from five metropolitan areas (n = 757), (3) a sample of siblings of MIDUS-1 participants (n = 950), and (4) a national twin sample (n = 1914). Participants were noninstitutionalized, English-speaking adults aged 25–74. A follow-up study (MIDUS-2) was conducted in 2004–2006, with 4963 participants successfully completing interviews. Additionally, a new Milwaukee African American oversample (n = 592) was recruited to increase racial diversity in the study. The Biomarker Project, initiated during MIDUS-2, included a subsample of participants (n = 1255) who completed the core survey and agreed to an overnight visit at one of the following three clinical research sites: Georgetown University; the University of California, Los Angeles; or the University of Wisconsin-Madison. During these visits, participants underwent physical examination, provided details of their medical history, and contributed fasting blood samples (collected prior to nicotine or caffeine consumption). Additional details on the biomarker protocol are available elsewhere [41]. All participating sites’ Institutional Review Boards approved data collection, and all participants provided informed consent.

Given the underrepresentation of non-White adults in the parent MIDUS study, we used two data sources in the present study. Data from the biomarker substudy were used to identify which positive psychological health measures were bivariately associated with CVH. We did not control for race in these analyses, given the low prevalence of non-White participants (e.g., 2.6% Black adults). Positive psychological health measures significantly associated with CVH in the biomarker substudy were then examined in the full MIDUS sample. Using the full MIDUS-2 sample, we then examined whether race was associated with differences in the positive psychological health measures.

### 2.2. Measures

#### 2.2.1. LE8

Cardiovascular health was assessed using the AHA’s validated LE8 components [1,2,42]. *Diet* was assessed using self-reported average daily or weekly consumption of fruits and vegetables, lean meat, ocean (oily) fish, fast food intake, beef or high fat intake, whole grains, and non-meat protein. Self-reported general moderate or vigorous *physical activity* in the home, work, or for leisure were collected; questions were augmented to reflect the season when data were collected (i.e., summer or winter engagement in activities). Self-reported *nicotine exposure* was assessed by asking participants’ current smoking status or age when the individual last regularly smoked for former smokers. Self-reported *sleep health* was quantified as the hours of sleep on weekdays and weekends. *BMI* was calculated using a participant’s weight and height measures collected during the biomarker substudy. *Blood lipids* were calculated by subtracting the blood high-density lipoprotein (HDL) from the total blood cholesterol level to obtain total non-HDL cholesterol. *Blood glucose* was assessed using the following two data sources: participants indicated whether they have or were diagnosed with diabetes, and blood fasting glucose levels. *Blood pressure* was assessed during the biomarker substudy and reflected one’s systolic and diastolic blood pressure. All factors were rescored to reflect a 0–100-point scale, with higher scores reflecting better health. LE8 scores reflect the average of an individual’s points across the eight domains. See Appendix A: Online Resource 1 for details on the method the data were transformed to LE8 scores.

#### 2.2.2. Negative Psychological Health Factors

*Depressive symptoms* were measured using the Center for Epidemiologic Studies-Depression Scale [43]. *Stressor reactivity* was assessed using the three-item stress reactivity subscale from the Multidimensional Personality Questionnaire, where participants were asked about their general response to everyday stressful events [44]. *Aggression* was measured using the four-item aggression subscale from the Multidimensional Personality Questionnaire; participants were asked about their enjoyment and tendency toward physical or emotional aggression [44]. *Pessimism* was measured using the Life Orientation Test’s three-item pessimism toward life subscale [45]. *Perceived stress* was assessed using the Perceived Stress Scale [46]. Trait anxiety was assessed using the Spielberger Trait Anxiety Inventory [47].

#### 2.2.3. Positive Psychological Health Factors

*Psychological well-being* was measured using the 42-item Ryff Psychological Well-Being Scale [48], as well as the Multidimensional Personality Scale (MPS) [44]. The seven-item life purpose subscale from the Ryff Psychological Well-Being scale was also used to measure *purpose in life*. *Mindfulness* was assessed using a nine-item mindfulness subscale developed by the MIDUS authors. Participants were asked to rate to what extent they are open and aware of the events and people around them. *Gratitude* was measured using a single-item question as part of a MIDUS-created religiosity questionnaire about the frequency one experiences a “deep sense of appreciation”. Both mindfulness and gratitude measures were derived from the same religiosity questionnaire developed for MIDUS, with mindfulness reflecting efforts to be more present and intentional, often associated with religious or spiritual beliefs. These items, though derived from religiosity items, reflect broader psychological constructs that transcend religious frameworks. Mindfulness, commonly practiced in religious traditions like Buddhism, is linked to spiritual awareness and presence [49]. Gratitude has long been central to religious practices, particularly in Christianity, as a way of acknowledging the divine and promoting well-being [50]. These constructs have been shown to promote mental health and well-being even in non-religious contexts, functioning as secular practices that foster psychological resilience and life satisfaction [51]. *Optimism* was measured using the Life Orientation Test’s three-item optimism toward life scale [45].

#### 2.2.4. Covariates

Covariates were selected based on their potential for confounding the association between race, CVH, and the psychological health correlates. Demographic covariates were age, gender (0 = man; 1 = woman), and sexual orientation (heterosexual vs. sexual minority). Socioeconomic covariates included education (0 = less than high school; 1 = high school diploma; 2 = college degree; 3 = some postsecondary education); one’s self-reported social standing in their community using the MacArthur Scale of Subjective Social Status [52]; and total household income (US$) from wage, pension, social security, and other sources.

### 2.3. Analytic Approach

Descriptive statistics were calculated to characterize the LE8, negative and positive psychological health factors, and sociodemographic aspects of the sample. Differences in these factors by race were examined using independent samples *t*-tests or chi-square tests as appropriate. If there were differences by race, we then tested whether differences in the positive psychological health measure persisted after sociodemographic adjustment using multiple regression. Data were analyzed using R, version 4.2.2. Significance for all tests was set to *p* < 0.05 for two-tailed tests. Unless otherwise stated, the presented estimates are unstandardized.

## 3. Results

### 3.1. Aim 1: Associations Between Psychological Health, LE8 (MIDUS Biomarker Substudy)

#### 3.1.1. Sample Characteristics

There were 1255 participants with valid data included in this analysis. The average age was 55.3 (SD = 11.8), 54.7% of whom were women (n = 577), 2.6% were Black adults (n = 27), and most had at least a high school diploma (96.5%). See Table 1 for additional details.

#### 3.1.2. Negative Psychological Health Factors

There was a significant bivariate association between the LE8 total score and the following negative psychological health factors: depressive symptoms (est. = −1.1, *p* < 0.001), stress reactivity (est. = −0.477, *p* = 0.046), pessimism (est. = −1.0, *p* < 0.001), perceived stress (est. = −0.27, *p* < 0.001), and trait anxiety (est. = −0.23, *p* < 0.001). After covariate adjustment, the relationship with depressive symptoms (est. = −1.20, *p* < 0.001), pessimism (est. = −0.81, *p* < 0.001), perceived stress (est. = −0.25, *p* = 0.005), and trait anxiety (est. = −0.19, *p* = 0.001) was attenuated but remained significant such that higher levels (i.e., greater negative psychological health) were associated with poorer overall CVH. Aggression was not associated with the LE8 total scores in either analysis.

There was a significant bivariate association between the LE8 health behavior subscore and all the following negative psychological health factors: depressive symptoms (est. = −2.20, *p* < 0.001), stress reactivity (est. = −1.10, *p* < 0.001), aggression (est. = −1.40, *p* = 0.001), pessimism (est. = −1.80, *p* < 0.001), perceived stress (est. = −0.49, *p* < 0.001), and trait anxiety (est. = −0.43, *p* < 0.001). After covariate adjustment, the relationship with depressive symptoms (est. = −1.90, *p* < 0.001), pessimism (est. = −1.20, *p* < 0.001), perceived stress (est. = −0.32, *p* = 0.008), and trait anxiety (est. = −0.29, *p* < 0.001) was attenuated but remained significant such that higher levels were associated with poorer LE8 health behavior subscores. Lastly, there were no significant bivariate or covariate-adjusted relationships between any negative psychological health factors and the LE8 health factor subscores (Table 2).

#### 3.1.3. Positive Psychological Health Factors

There was a significant bivariate association between the LE8 total score and the following positive psychological health factors: Ryff’s psychological well-being (est. = 0.06, *p* < 0.001), purpose in life (est. = 0.44, *p* < 0.001), and optimism (est. = 0.75, *p* < 0.001). After covariate adjustment, all relationships remained significant (Ryff’s psychological well-being est. = 0.05, *p* = 0.005; purpose in life est. = 0.36, *p* < 0.001; optimism est. = 0.75, *p* = 0.009) whereby a higher level (i.e., greater positive psychological health) was associated with better overall CVH. There were no significant association between MPS psychological well-being, mindfulness, or gratitude and LE8 total scores in either analysis.

There was a significant bivariate association between the LE8 health behavior subscore and the following positive psychological health factors: Ryff’s psychological well-being (est. = 0.13, *p* < 0.001), MPS psychological well-being (est. = 1.30, *p* = 0.001), purpose in life (est. = 0.71, *p* < 0.001), gratitude (overall *p* = 0.032), and optimism (est. = 1.50, *p* < 0.001). After covariate adjustment, a significant relationship persisted for Ryff’s psychological well-being (est. = 0.07, *p* = 0.002), purpose in life (est. = 0.48, *p* < 0.001), and optimism (est. = 0.87, *p* = 0.008) such that greater positive psychological health was associated with better health behavior scores. There was no significant association with mindfulness in either analysis.

There was a significant bivariate association between LE8 health factor subscores with MPS psychological well-being in an unexpected direction; greater well-being was associated with a poorer health factor subscore (est. = −0.46, *p* = 0.02). This relationship was not significant after covariate adjustment (est. = −0.12, *p* = 0.70). In covariate-adjusted analyses, the relationship with purpose in life became significant such that greater purpose was associated with a better health factor subscore. Ryff’s psychological well-being, mindfulness, gratitude, and optimism were not significantly associated with health factor subscores in either analysis (Table 2).

### 3.2. Aim 2: Racial Differences in Psychological Health Factors Associated with LE8 (MIDUS Parent Study)

#### 3.2.1. Sample Characteristics

There were 4702 participants with valid data included in this analysis. The average age was 55.5 (SD = 12.5), 53.4% of whom were women (n = 2512), 4.9% Black adults (n = 229), and most had at least a high school diploma (94.0%). See Table 1 for additional details.

#### 3.2.2. Negative Psychological Health Factors

Among the negative psychological health factors, only pessimism had significant differences by race (Black > White; *p* < 0.001). There were no significant differences by race in depressive symptoms (*p* = 0.270), stress reactivity (*p* = 0.153), aggression (*p* = 0.066), perceived stress (*p* = 0.480), or trait anxiety (*p* = 0.433; Table 3).

#### 3.2.3. Positive Psychological Health Factors

There were significant differences by race in the following positive psychological health factors: MPS psychological well-being (Black > White; *p* < 0.001), gratitude (Black > White, overall *p* < 0.001), and optimism (Black > White; *p* < 0.001). There were no differences by race in Ryff’s psychological well-being (*p* = 0.652) or purpose in life (*p* = 0.870). Although gratitude was not associated with any LE8 measure, there were significant differences by race (Black > White, *p* < 0.001; Table 3).

## 4. Discussion

In this cross-sectional analysis, using data from the MIDUS study, we observed a low prevalence of ideal CVH that was comparable to other cohort-based studies in the United States [53]. Overall CVH and CVH behaviors were bivariately associated with less negative psychological health and greater positive psychological health, and these associations were generally robust to covariate adjustment. This pattern of results was not observed when considering CVH factors alone, being largely unrelated to either negative or positive psychological health factors. Together, these results support existing findings that psychological health influences CVH through health behaviors [32]. It is possible that associations between negative and positive psychological health factors with CVH factors vary over time [31,54] or differ as a function of age [55] and should be explored in future work.

We found that most of the negative psychological health factors were associated with overall CVH and CVH behaviors but not CVH factors, even after controlling for sociodemographic characteristics. This extends previous work that found negative psychological health was associated with specific cardiovascular diseases [33] and suggests that interventions targeting negative psychological health may also improve CVH [56]. Interestingly, pessimism was the only negative psychological health factor that was both related to different CVH outcomes and demonstrated significant Black–White racial differences. The finding that Black participants had higher levels of pessimism aligns with the racial weathering hypothesis, which posits that chronic exposure to discrimination and socioeconomic adversity may contribute to accelerated physiological deterioration and negative psychological states [57,58]. While some research suggests that cultural strengths, such as collectivism and spirituality, promote resilience [59,60], the cumulative impact of structural racism may nonetheless contribute to elevated stress and pessimism. Although Black adults have greater stress exposure compared to White adults, they may appraise these experiences as less stressful [61,62]. In addition to cognitively reappraising stressors, Black adults may have developed adaptive coping strategies to reduce the adverse consequences of discrimination on health, as evinced by coping mechanisms tied to spirituality and social support, which may act as protective factors [63,64]. Beyond these stress appraisal and coping mechanisms, we also observed significant racial differences in positive psychological health. Specifically, Black adults reported higher optimism and gratitude compared to White adults, aligning with prior research on resilience-oriented traits in marginalized populations. This pattern may reflect the role of culturally embedded coping strategies, including spirituality, collectivism, and social support, in promoting well-being despite systemic adversity. However, the finding that pessimism was also higher in Black adults suggests that stress exposure remains a salient factor influencing psychological health outcomes. Incorporating cultural and socio-historical context into the interpretation of optimism and pessimism is crucial, as these constructs may have different manifestations and health implications depending on one’s lived experience and coping strategies, particularly in marginalized groups. To reduce racial CVH disparities, interventions should incorporate both psychological and structural components. Programs that promote stress resilience (e.g., mindfulness and cognitive-behavioral strategies) alongside culturally tailored health education may be particularly effective. Additionally, integrating positive psychology interventions, such as gratitude and purpose-in-life exercises, into CVH programs could enhance adherence to heart-healthy behaviors. Negative psychological health factors, such as pessimism and perceived stress, may contribute to poor CVH through both physiological (e.g., heightened inflammation and dysregulated HPA axis) and behavioral pathways (e.g., reduced engagement in health-promoting behaviors and increased cardiovascular risk behaviors). Prior research has shown that chronic stress and depression are linked to increased allostatic load, which can negatively impact cardiovascular function over time [65,66,67,68]. Future studies should investigate whether these psychological health differences translate into differential CVH outcomes and whether interventions targeting both risk and resilience factors can help mitigate disparities. While psychological factors contribute to CVH disparities, they do not fully explain the persistent racial differences observed. Future research should explore additional mechanisms, such as structural barriers to healthcare, environmental stressors (e.g., neighborhood safety and food access), and socioeconomic disparities, which may also play a crucial role in shaping cardiovascular health outcomes. Future research should explore these relationships among a diverse sample of US adults.

Regarding positive psychological health factors, we found that psychological well-being, optimism, and gratitude were both related to better overall CVH and higher engagement in CVH-enhancing behaviors. These factors were also higher in Black relative to White adults. These results complement the existing work that uses a combination of positive psychology programs and/or motivational interviewing to improve CVH behaviors [69,70,71]. However, such interventions may not be equally beneficial for all adults, as the positive psychology-based TRIUMPH intervention did not lead to blood pressure control, especially among Black adults with higher baseline depressive symptoms or perceived stress [72]. Thus, it is likely that positive psychology-based interventions may need an adjunct therapy to maximize CVH promotion and reduce disparities. For example, interventions with multiple as opposed to one positive psychology exercise have greater psychological health benefits among those with CVD [73]. Multilevel interventions meant to improve community/neighborhood positive psychological health may be another approach to better improve CVH while also attending to its upstream social determinants [74]. Positive psychological factors, such as optimism and psychological well-being, may serve as protective factors against cardiovascular disease by fostering resilience and adaptive coping mechanisms. Research suggests that individuals with higher optimism are more likely to engage in regular physical activity and maintain healthy diets, both of which contribute to better CVH [75,76,77]. More research is warranted to identify to what extent positive psychology-based interventions could complement other CVH promotion interventions.

### Limitations and Future Directions

This study focused on individual-level psychological health; however, broader social and structural factors, including economic and environmental conditions, also shape CVH [78,79,80,81]. Recognizing this, the AHA recently recommended the comprehensive data collection of the social determinants of health [82]. A more holistic understanding of the myriad upstream contributors to CVH—from the cell to the society—will facilitate the development of multilevel interventions that could improve health and reduce disparities [83] over unilevel, largely individual-based interventions. While this study provides important insights, a key limitation is the lack of broader racial and ethnic diversity in the sample. Future studies should prioritize recruitment strategies that ensure a more inclusive representation of racial and ethnic groups. Employing targeted outreach and community-engaged research approaches may enhance the generalizability of findings and lead to a more comprehensive understanding of the intersection between psychological health and CVH disparities. Additionally, future research should explore how social and structural determinants interact with psychological health to influence CVH across diverse populations.

Regarding the measurement of gratitude, a single-item gratitude measure was used due to its feasibility in large-scale, population-based studies. While this approach is efficient, it is also a limitation, as a single-item measure may not capture the full complexity of the construct. Single-item measures of psychological constructs have been shown to be reliable and valid in certain contexts, but they may lack the depth and specificity offered by multi-item scales, which could provide a more nuanced understanding of this psychological factor. Future research may benefit from employing multi-item measures of gratitude to better capture its multifaceted nature. Additionally, self-reported measures used to assess psychological health (e.g., depressive symptoms, stress reactivity, and well-being) may be subject to various biases, including social desirability bias and recall bias. To mitigate these, we adjusted for potential confounders in our models to account for variability across demographic groups. Future research should consider comprehensive assessments of psychological health to further control for the impact of self-report biases.

Future research should also examine the mechanisms by which psychological resilience, such as optimism, gratitude, and purpose in life, influences CVH outcomes across different racial and ethnic groups. This could include investigating how cultural factors and coping mechanisms may shape psychological resilience and its association with CVH. Longitudinal studies would be particularly valuable in understanding how changes in psychological health over the lifespan contribute to disparities in CVH, especially during midlife, a critical period for CVH deterioration. Moreover, multilevel interventions that target both individual psychological health and broader social determinants of health should be explored to mitigate disparities and improve overall cardiovascular health.

An additional limitation of this study is that LE8 was only calculated for a subset of participants. This was due to the availability of specific LE8 components, such as objective cardiometabolic measures, which were only collected in the MIDUS Biomarker Project. As a result, our analytic sample was restricted to individuals who participated in this substudy. This may limit the generalizability of findings to the broader MIDUS cohort. Future research should examine strategies to estimate LE8 with more diverse samples.

## 5. Conclusions

In conclusion, there is growing epidemiologic support demonstrating the association between psychological and cardiovascular health outcomes [36,38,84]. Interventions targeting psychological health appear beneficial for CVH [33], but it is unclear which factors should be targeted to maximally enhance CVH. These results suggest that both reducing negative and positive psychological health may positively affect overall CVH and CVH behaviors. These results also suggest that Black–White differences in CVH are not largely explained by racial differences in the psychological health measures included in this study. Future research should continue examining which modifiable factors drive CVH disparities to develop interventions to eliminate disparities and improve CVH for all.

## Figures and Tables

**Table 1:** Participant characteristics: mean (standard deviation) or n (valid %).

	Biomarker Substudy (Aim 1) n = 1255	Parent Study (Aim 2) n = 4702
Demographic Covariates		
Age	55.3 (11.8)	55.5 (12.5)
Gender		
Women	201 (54.7%)	2512 (53.4%)
Men	477 (45.3%)	2190 (46.6%)
Race		
Black	27 (2.6%)	229 (4.9%)
White	996 (96.8%)	4493 (95.1%)
Education		
<High School Diploma	37 (3.5%)	281 (6.0%)
High School Diploma	441 (42.0%)	2302 (49.0%)
Some Postsecondary Education	324 (30.8%)	1278 (27.2%)
College Degree	249 (23.7%)	834 (17.8%)
Social Standing in Community	4.4 (1.7)	4.5 (1.8)
Household Income	$76,672.40 ($60,409.20)	$71,614.8 ($60,741.5)
LE8		
Total Score	65.9 (13.7)	
Health Behavior Subscore	65.7 (19.7)	
Diet Quality	48.3 (32.6)	
Physical Activity	60.1 (47.2)	
Nicotine Exposure	63.4 (33.2)	
Sleep Health	86.0 (22.7)	
Health Factor Subscore	65.0 (17.4)	
BMI	58.6 (33.9)	
Blood Lipids	71.0 (29.3)	
Blood Glucose	68.0 (25.3)	
Blood Pressure	56.4 (30.3)	
Negative Psychological Health Factors		
Depressive Symptoms	0.6 (1.7)	0.5 (1.6)
Stress Reactivity	6.1 (2.3)	6.2 (2.2)
Aggression	5.4 (1.7)	5.4 (1.8)
Pessimism	6.1 (2.9)	6.6 (3.1)
Perceived Stress	22.2 (6.3)	21.6 (6.2)
Trait Anxiety	34.3 (9.1)	33.4 (8.7)
Positive Psychological Health Factors		
Psychological Well-Being (Ryff)	235.2 (33.8)	231.2 (35.0)
Psychological Well-Being (Multidimensional Personality Scale)	9.1 (1.7)	9.0 (1.8)
Purpose in Life	39.6 (6.5)	38.5 (7.0)
Mindfulness	34.2 (6.3)	34.0 (6.1)
Gratitude		
Never	27 (2.6%)	100 (2.6%)
Rarely	79 (7.5%)	329 (8.7%)
Sometimes	458 (43.7%)	1696 (44.8%)
Often	485 (46.2%)	1660 (43.9%)
Optimism	12.0 (2.4)	11.8 (2.5)

**Table 2:** Unstandardized associations (95% CI, *p*-value) between hypothesized psychosocial risk and resilience factors with LE8 (Aim 1; n = 1255).

	LE8 Total Score		Health Behavior Subscore		Health Factor Subscore	
	Step 1	Step 2	Step 1	Step 2	Step 1	Step 2
Negative Psychological Health Factors						
Depressive Symptoms	**−1.1 (95% CI: −1.7, −0.52, *p* < 0.001)**	**−1.2 (95% CI: −1.8, −0.58, *p* < 0.001)**	**−2.2 (95% CI: −3.0, −1.5, *p* < 0.001)**	**−1.9 (95% CI: −2.7, −1.1, *p* < 0.001)**	−0.18 (95% CI: −0.84, 0.47, *p* = 0.60)	−0.63 (95% CI: −1.3, 0.03, *p* = 0.061)
Stress Reactivity	**−0.47 (95% CI: −0.93, −0.01, *p* = 0.046)**	−0.36 (95% CI: −0.84, 0.12, *p* = 0.14)	**−1.1 (95% CI: −1.7, −0.47, *p* < 0.001)**	−0.50 (95% CI: −1.2, 0.16, *p* = 0.14)	0.08 (95% CI: −0.40, 0.57, *p* = 0.70)	−0.26 (95% CI: −0.77, 0.25, *p* = 0.30)
Aggression	−0.52 (95% CI: −1.1, 0.07, *p* = 0.083)	−0.22 (95% CI: −0.82, 0.38, *p* = 0.50)	**−1.4 (95% CI: −2.2, −0.55, *p* = 0.001)**	−0.80 (95% CI: −1.6, 0.04, *p* = 0.062)	−0.19 (95% CI: −0.82, 0.45, *p* = 0.60)	−0.11 (95% CI: −0.75, 0.54, *p* = 0.70)
Pessimism	**−1.0 (95% CI: −1.3, −0.68, *p* < 0.001)**	**−0.81 (95% CI: −1.2, −0.45, *p* < 0.001)**	**−1.8 (95% CI: −2.2, −1.3, *p* < 0.001)**	**−1.2 (95% CI: −1.7, −0.67, *p* < 0.001)**	−0.13 (95% CI: −0.50, 0.24, *p* = 0.50)	−0.20 (95% CI: −0.60, 0.20, *p* = 0.30)
Perceived Stress	**−0.27 (95% CI: −0.44, −0.11, *p* = 0.001)**	**−0.24 (95% CI: −0.41, −0.07, *p* = 0.005)**	**−0.49 (95% CI: −0.72, −0.26, *p* < 0.001)**	**−0.32 (95% CI: −0.55, −0.08, *p* = 0.008)**	−0.05 (95% CI: −0.23, 0.13, *p* = 0.60)	−0.15 (95% CI: −0.34, 0.03, *p* = 0.10)
Trait Anxiety	**−0.23 (95% CI: −0.34, −0.11, *p* < 0.001)**	**−0.19 (95% CI: −0.31, −0.08, *p* = 0.001)**	**−0.43 (95% CI: −0.58, −0.27, *p* < 0.001)**	**−0.29 (95% CI: −0.46, −0.12, *p* < 0.001)**	−0.03 (95% CI: −0.15, 0.09, *p* = 0.60)	−0.09 (95% CI: −0.22, 0.04, *p* = 0.20)
Positive Psychological Health Factors						
Psychological Well-Being (Ryff)	**0.06 (95% CI: 0.03, 0.09, *p* < 0.001)**	**0.05 (95% CI: 0.02, 0.08, *p* = 0.005)**	**0.13 (95% CI: 0.09, 0.17, *p* < 0.001)**	**0.07 (95% CI: 0.03, 0.12, *p* = 0.002)**	0.00 (95% CI: −0.03, 0.03, *p* > 0.9)	0.02 (95% CI: −0.01, 0.06, *p* = 0.20)
Psychological Well-Being (Multidimensional Personality Scale)	0.51 (95% CI: −0.08, 1.1, *p* = 0.092)	0.24 (95% CI: −0.39, 0.87, *p* = 0.50)	**1.3 (95% CI: 0.53, 2.2, *p* = 0.001)**	0.56 (95% CI: −0.30, 1.4, *p* = 0.20)	−0.46 (95% CI: −1.1, 0.17, *p* = 0.20)	−0.12 (95% CI: −0.79, 0.55, *p* = 0.70)
Purpose in Life	**0.44 (95% CI: 0.29, 0.60, *p* < 0.001)**	**0.36 (95% CI: 0.19, 0.53, *p* < 0.001)**	**0.71 (95% CI: 0.50, 0.92, *p* < 0.001)**	**0.48 (95% CI: 0.24, 0.72, *p* < 0.001)**	0.14 (95% CI: −0.03, 0.31, *p* = 0.10)	**0.19 (95% CI: 0.01, 0.37, *p* = 0.041)**
Mindfulness	−0.03 (95% CI: −0.20, 0.13, *p* = 0.70)	−0.04 (95% CI: −0.21, 0.12, *p* = 0.60)	0.10 (95% CI: −0.12, 0.33, *p* = 0.40)	0.06 (95% CI: −0.17, 0.29, *p* = 0.60)	−0.10 (95% CI: −0.28, 0.08, *p* = 0.30)	−0.10 (95% CI: −0.28, 0.08, *p* = 0.30)
Gratitude (Never = Ref)						
Rarely	4.0 (−3.0, 11)	2.9 (−3.8, 9.7)	**7.8 (−2.2, 18)**	6.7 (−3.0, 16)	−0.53 (−8.2, 7.1)	−1.4 (−8.8, 6.1)
Sometimes	4.0 (−3.0, 11)	3.2 (−2.9, 9.3)	**7.7 (−1.4, 17)**	5.1 (−3.6, 14)	0.25 (−6.6, 7.1)	0.06 (−6.5, 6.6)
Often	5.8 (−0.57, 12)	3.8 (−2.3, 10)	**11 (1.8, 20)**	6.5 (−2.4, 15)	−0.57 (−7.4, 6.2)	−0.59 (−7.2, 6.0)
	Overall *p* = 0.2	Overall *p* = 0.06	**Overall *p* = 0.032**	Overall *p* = 0.40	Overall *p* > 0.90	Overall *p* = 0.9
Optimism	**0.75 (95% CI: 0.32, 1.2, *p* < 0.001)**	**0.60 (95% CI: 0.15, 1.1, *p* = 0.009)**	**1.5 (95% CI: 0.86, 2.1, *p* < 0.001)**	**0.87 (95% CI: 0.23, 1.5, *p* = 0.008)**	−0.01 (95% CI: −0.47, 0.45, *p* > 0.9)	0.18 (95% CI: −0.31, 0.67, *p* = 0.5)

Note. Step 1 involves the unadjusted, bivariate associations between the variables of interest. Step 2 adjusts for age, gender, sexual orientation, education, social standing, and household income; there were too few non-White participants to adjust for race in this analysis. After covariate adjustment, more depressive symptoms, higher pessimism, higher stress, and higher trait anxiety were associated with poorer LE8 total scores and health behavior subscores. After covariate adjustment, greater psychological well-being (Ryff), purpose in life, and optimism were associated with better LE8 total scores and health behavior subscores. Greater purpose in life was also associated with better health factor subscores. Bolded values indicate associations with *p* < 0.05.

**Table 3:** Black–White differences among psychosocial risk and protective factors bivariately associated with LE8 (Aim 2; n = 4702).

	Black Adults (n = 229) M (SD) or n (%)	White Adults (n = 4473) M (SD) or n (%)	*p*-Value
Negative Psychological Health Factors			
Depressive Symptoms	0.4 (1.5)	0.5 (1.7)	0.270
Stress Reactivity	6.5 (2.4)	6.2 (2.2)	0.153
Aggression	5.9 (2.4)	5.4 (1.8)	0.066
Pessimism	**7.8 (3.8)**	**6.6 (3.0)**	**<0.001**
Perceived Stress	22.6 (7.0)	21.6 (6.1)	0.480
Trait Anxiety	35.6 (11.1)	33.4 (8.7)	0.433
Positive Psychological Health Factors			
Psychological Well-Being (Ryff)	229.7 (36.2)	231.2 (34.9)	0.652
Psychological Well-Being (Multidimensional Personality Scale)	**9.5 (1.8)**	**9.0 (1.8)**	**<0.001**
Purpose in Life	38.4 (7.6)	38.5 (6.9)	0.870
Gratitude			**<0.001**
Never	**0 (0%)**	**100 (2.7%)**	
Rarely	**11 (7.4%)**	**318 (8.7%)**	
Sometimes	**46 (31.1%)**	**1650 (45.4%)**	
Often	**91 (61.5%)**	**1569 (43.1%)**	
Optimism	**12.6 (2.4)**	**11.8 (2.5)**	**<0.001**

Note. Mindfulness was not bivariately associated with either the LE8 total score or subscores and was omitted from this analysis. Compared to White participants, Black participants reported higher pessimism, psychological well-being (Multidimensional Personality Scale), gratitude, and optimism. Bolded values indicate associations with *p* < 0.05.

## Data Availability

The MIDUS data used for this study are publicly available through the National Archive of Computerized Data on Aging and can be retrieved from https://www.icpsr.umich.edu/web/pages/NACDA/midus.html (accesses on 12 December 2022).

## Abbreviations

AHA	American Heart Association
BMI	Body Mass Index
CI	Confidence Interval
CVH	Cardiovascular Health
CVD	Cardiovascular Disease
HbA1c	Hemoglobin A1c
IRB	Institutional Review Board
LE8	Life’s Essential 8
MIDUS	Midlife in the United States Study
MPS	Multidimensional Personality Scale
PWB	Psychological Well-Being

## Appendix A

Online Resource 1. Calculating life’s essential 8 scores.

**Transformed Variable Name**	**Original Variable Scoring**	**How to Calculate Life’s Essential 8 Scores**
Life’s Essential 8 Average Score (Outcome of interest)	Scores are continuous and range from 0 to 100.	Average of Diet_Points, Nicotine_Points, MVPA_Points, BMI_Points, Non-HDL_Points, Glucose_Points, BP_Points, and Sleep_Points.
Life’s Essential 8 Behavioral Subscore (Outcome of interest)	Scores should be continuous and range from 0 to 100	Average of Diet_Points, Nicotine_Points, MVPA_Points, and Sleep_Points.
Life’s Essential 8 Health Factor Subscore (Outcome of interest)	Scores should be continuous and range from 0 to 100	Average of BMI_Points, Non-HDL_Points, Glucose_Points, and BP_Points.
Diet Quality Subscore	For fruit/vegs, whole grain, answers reflecting daily servings. 1 = None 2 = 1–2 servings/day 3 = 3–4 servings/day 4 = 5 or more servings/day 5 = Less than 1 serving/day 7 = Don’t know (missing) 8 = Missing 9 = Inapplicable (missing) For oily fish, fast food, beef/high fat answers reflecting weekly servings. 1 = Never 2 = Less than once/week 3 = 1–2x/week 4 = 3–4x/week 5 = 5 or more x/week 7 = Don’t know (missing) 8 = Missing 9 = Inapplicable For B4H33: 1 = Yes 2 = No 7 = Don’t know (missing) 8 = Missing 9 = Inapplicable (missing) For B4H34: 1 = Everyday 2 = 5 or 6 days/week 3 = 3 or 4 days/week 4 = 1 or 2 days/week 5 = less than 1 day/week 6 = Never drinks 7 = Don’t know (missing) 8 = Missing 9 = Inapplicable (missing) For B4H36: Continuous variable of number of drinks of standard unit of alcohol consumed. 97 = Don’t know (missing) 98 =Missing 99 = Inapplicable (missing)	To calculate the individual diet components, recode the variables using the following. These variables will be binary yes (1)/no (0) to indicate whether they are eating the sufficient amount of that item to count toward a high-quality diet as follows: Fruit/Vegetables: If participant reports eating at least 3 servings/day (i.e., either 3 or 4 for B4H21), then FruitVeg_Quality = 1. Responses of 1, 2, and 5 receive a FruitVeg_Quality = 0. Whole Grains: If participant reports eating at least 3 servings/day (i.e., either 3 or 4 for B4H22), then Grain_Quality = 1. Responses of 1, 2, and 5 receive a Grain_Quality = 0. Oily Fish: If participant reports eating at least 1 serving/week (i.e., Responses of 3, 4, or 5 for B4H23A), then Fish_Quality = 1. Responses of 1 or 2 receive a Fish_Quality = 0. Fast Food: If participant reports eating out less than once per week (i.e., either 1 or 2 for B4H24), then Fast_Food_Quality = 1. Responses of 3, 4, and 5 receive a Fast_Food_Quality = 0. Beef/High Fat: If participant reports eating beef/high fat less than 3 times a week (i.e., either 1, 2, or 3 for B4H23B), then High_Fat_Quality = 1. Responses of 4 or 5 receive a High_Fat_Quality = 0. Alcohol Consumption for Men: If participant reported drinking between 1 and 2 drinks for B4H36 (regardless of how they scored for B4H34), they receive an Alcohol_Quality = 1. Any other quantity (regardless of B4H34 value) receives an Alcohol_Quality = 0. Alcohol Consumption for Women: If participant reported drinking 1 or fewer drinks for B4H36 (regardless of how they scored for B4H34), they receive an Alcohol_Quality = 1. Any other quantity (regardless of B4H34 value) receives an Alcohol_Quality = 0. Any participant (regardless of gender) reporting No for B4H33 receives an Alcohol_Quality = 0. If participant reports “Yes” for B4H33 but reports “never drinking” (i.e., 6) for B4H34 and a 0 for B4H36, they are also coded as Alcohol_Quality = 0. To calculate Diet_Points: First, add FruitVeg_Quality, Grain_Quality, Fish_Quality, Fast_Food_Quality, High_Fat_Quality, and Alcohol_Quality to obtain a Total_Diet_Quality score that could range from 0 to 6. Using these values, if Total_Diet_Quality is … Between 5 and 6, then Diet_Points = 100; 4, then Diet_Points = 80; 3, then Diet_Points = 50; 2, then Diet_Points = 25; 1 or 0, then Diet_Points = 0.
Physical Activity Subscore	1 = Yes 2 = No 7 = Don’t Know 8 = Missing 9 = Inapplicable	If participant answered “Yes”, calculate the number of minutes/week they engage in moderate and vigorous physical activity. If participant answered “No”, participant was coded as “inapplicable” for branching intensity questions. A “No” participant would receive a score of 0 points for the MVPA_Points score.
	Continuous values quantifying engagement in specific activity 97 = Don’t Know (Missing) 98 = Missing 99 = Inapplicable	Activity A: Multiply the three values together to obtain the total number of minutes/week participant engages in this activity.
	1 = Vigorous 2 = Moderate 3 = Light 7 = Don’t Know (missing) 8 = Missing 9 = Inapplicable (Missing)	Activity A: If participant reports this activity as either vigorous or moderate, the total number of minutes would be added to their moderate/vigorous physical activity. IF THE ACTIVITY IS VIGOROUS, THIS VALUE SHOULD BE DOUBLED (For example, if participant has 450 min/week in an activity they called “vigorous”, they would have 900 min total for this activity. If the activity is reported as moderate, then they would have 450 min/week of this activity). If the activity is light, do not count this activity toward their physical activity score.
	Continuous values quantifying engagement in specific activity 97 = Don’t Know (Missing) 98 = Missing 99 = Inapplicable	Activity B: Multiply the three values together to obtain the total number of minutes/week participant engages in this activity.
	1 = Vigorous 2 = Moderate 3 = Light 7 = Don’t Know (missing) 8 = Missing 9 = Inapplicable (Missing)	Activity B: If participant reports this activity as either vigorous or moderate, the total number of minutes would be added to their moderate/vigorous physical activity. IF THE ACTIVITY IS VIGOROUS, THIS VALUE SHOULD BE DOUBLED (For example, if participant has 450 min/week in an activity they called “vigorous”, they would have 900 min total for this activity. If the activity is reported as moderate, then they would have 450 min/week of this activity). If the activity is light, do not count this activity toward their physical activity score.
	Continuous values quantifying engagement in specific activity 97 = Don’t Know (Missing) 98 = Missing 99 = Inapplicable	Activity C: Multiply the three values together to obtain the total number of minutes/week participant engages in this activity.
	1 = Vigorous 2 = Moderate 3 = Light 7 = Don’t Know (missing) 8 = Missing 9 = Inapplicable (Missing)	Activity C: I If participant reports this activity as either vigorous or moderate, the total number of minutes would be added to their moderate/vigorous physical activity. IF THE ACTIVITY IS VIGOROUS, THIS VALUE SHOULD BE DOUBLED (For example, if participant has 450 min/week in an activity they called “vigorous”, they would have 900 min total for this activity. If the activity is reported as moderate, then they would have 450 min/week of this activity). If the activity is light, do not count this activity toward their physical activity score.
	Continuous values quantifying engagement in specific activity 97 = Don’t Know (Missing) 98 = Missing 99 = Inapplicable	Activity D: Multiply the three values together to obtain the total number of minutes/week participant engages in this activity.
	1 = Vigorous 2 = Moderate 3 = Light 7 = Don’t Know (missing) 8 = Missing 9 = Inapplicable (Missing)	Activity D: If participant reports this activity as either vigorous or moderate, the total number of minutes would be added to their moderate/vigorous physical activity. IF THE ACTIVITY IS VIGOROUS, THIS VALUE SHOULD BE DOUBLED (For example, if participant has 450 min/week in an activity they called “vigorous”, they would have 900 min total for this activity. If the activity is reported as moderate, then they would have 450 min/week of this activity). If the activity is light, do not count this activity toward their physical activity score.
	Continuous values quantifying engagement in specific activity 97 = Don’t Know (Missing) 98 = Missing 99 = Inapplicable	Activity E: Multiply the three values together to obtain the total number of minutes/week participant engages in this activity.
	1 = Vigorous 2 = Moderate 3 = Light 7 = Don’t Know (missing) 8 = Missing 9 = Inapplicable (Missing)	Activity E: If participant reports this activity as either vigorous or moderate, the total number of minutes would be added to their moderate/vigorous physical activity. IF THE ACTIVITY IS VIGOROUS, THIS VALUE SHOULD BE DOUBLED (For example, if participant has 450 min/week in an activity they called “vigorous”, they would have 900 min total for this activity. If the activity is reported as moderate, then they would have 450 min/week of this activity). If the activity is light, do not count this activity toward their physical activity score.
	Continuous values quantifying engagement in specific activity 97 = Don’t Know (Missing) 98 = Missing 99 = Inapplicable	Activity F: Multiply the three values together to obtain the total number of minutes/week participant engages in this activity.
	1 = Vigorous 2 = Moderate 3 = Light 7 = Don’t Know (missing) 8 = Missing 9 = Inapplicable (Missing)	Activity F: If participant reports this activity as either vigorous or moderate, the total number of minutes would be added to their moderate/vigorous physical activity. IF THE ACTIVITY IS VIGOROUS, THIS VALUE SHOULD BE DOUBLED (For example, if participant has 450 min/week in an activity they called “vigorous”, they would have 900 min total for this activity. If the activity is reported as moderate, then they would have 450 min/week of this activity). If the activity is light, do not count this activity toward their physical activity score.
	Continuous values quantifying engagement in specific activity 97 = Don’t Know (Missing) 98 = Missing 99 = Inapplicable	Activity G: Multiply the three values together to obtain the total number of minutes/week participant engages in this activity.
	1 = Vigorous 2 = Moderate 3 = Light 7 = Don’t Know (missing) 8 = Missing 9 = Inapplicable (Missing)	Activity G: If participant reports this activity as either vigorous or moderate, the total number of minutes would be added to their moderate/vigorous physical activity. IF THE ACTIVITY IS VIGOROUS, THIS VALUE SHOULD BE DOUBLED (For example, if participant has 450 min/week in an activity they called “vigorous”, they would have 900 min total for this activity. If the activity is reported as moderate, then they would have 450 min/week of this activity). If the activity is light, do not count this activity toward their physical activity scores.
		Add MVPA_ActivityA, MVPA_ActivityB, MVPA_ActivityC, MVPA_ActivityD, MVPA_ActivityE, MVPA_ActivityF, and MVPA_ActivityG to obtain MVPA_ActivityTotal (will reflect total # of minutes of moderate and vigorous physical activity participant engages in during a week). Calculating MVPA_Points: If MVPA_ActivityTotal is … ≥150, then MVPA_Points = 100; Between 120 and 149, then MVPA_Points = 90; Between 90 and 119, then MVPA_Points = 80; Between 60 and 89, then MVPA_Points = 60; Between 30 and 59, then MVPA_Points = 40; Between 1 and 29, then MVPA_Points = 20; 0, then MVPA_Points = 0, Participants who answered “No” to B4H25 should receive an MVPA_Points score of 0.
Nicotine Exposure Subscore	A37→ continuous variable for age. 96 = Never had a cigarette (missing) 97 = Don’t know (missing) 98 = Refused 1 = Yes 2 = No	Individuals who have a nonmissing B1PA37 value AND indicated they are a current nonsmoker on the B1PA42 item are coded as “former smoker”. To determine how long ago they quit, perform the following: B1PRAGE_2019−B1PA42 to calculate the amount of time elapsed from the time they last smoked to their current age. For clarity, we will call this variable Years_Since_Nicotine_Exposure Calculating Nicotine_Points: If participant reported … Never having a cigarette (96) for B1PA37, then Nicotine_Points = 100; Being a former smoker and has ≥5 Years_Since_Nicotine_Exposure, then Nicotine_Points = 75; Being a former smoker and has between 1 and 4.9 [repeating] Years_Since_Nicotine_Exposure, then Nicotine_Points = 50; Being a former smoker and has <1 Years_Since_Nicotine_Exposure, then Nicotine_Points = 25; “Yes” (1) for B1PA39, then Nicotine_Points = 0. Subtract 20 points from Nicotine_Points (unless their score is 0) if participant indicates that someone smokes in their home (i.e., answered “yes” for B4H32).
Sleep Health Subscore	Numbers are continuous and reflect number of hours slept −1 = No questionnaire administered 98 = Refused	First, calculate (B1SA57A × 0.714) + (B1SA57B × 0.286) to find the average number of sleep hours during the week (for clarity, we will call this Sleep_Total). Calculating Sleep_Points: If participant has a Sleep_Total … Between 7.0 and 8.9 [repeating], then Sleep_Points = 100; Between 9.0 and 9.9 [repeating], then Sleep_Points = 90; Between 6.0 and 6.9 [repeating], then Sleep_Points = 70; Between 5.0 and 5.9 [repeating] OR ≥10.0, then Sleep_Points = 40; Between 4.0 and 4.9 [repeating], then Sleep_Points = 20; <4.0, then Sleep_Points = 0.
BMI Subscore	Numbers are continuous 997 = Don’t know (missing) 998 = Missing 999 = Inapplicable	Calculating BMI points: If participant has a BMI value … <25, BMI_Points = 100; Between 25.0 and 29.9, BMI_Points = 75; Between 30.0 and 34.9, BMI_Points = 30; Between 35.0 and 39.9, BMI_Points = 15; ≥40.0, BMI_Points = 0.
Blood Lipids Subscore	Values are continuous and in mg/dL 998 = Missing 999 = Inapplicable (missing) 1 = Daily 2 = A few times/week 3 = Once/week 4 = A few times/month 5 = Once this month −1 = Does not have questionnaire (missing) 8 = Refused 9 = Inappropriate	B4BCHOL−B4BHDL to obtain total non-HDL cholesterol Calculating Non-HDL_Points: If participant has a non-HDL cholesterol of … <130, then Non-HDL_Points = 100; 130–159, then Non-HDL_Points = 60; 160–189, then Non-HDL_Points = 40; 190–219, then Non-HDL_Points = 20; ≥220, then Non-HDL_Points = 0. If participant endorsed items 1–5 for B1SA12CY, subtract 20 points from Non-HDL_Points score.
Blood Glucose Subscore	Values are a percent of HbA1C 98 = Missing 99 = Inapplicable 1 = Yes 2 = No 3 = Borderline (B4H1I only) 7 = Don’t Know 8 = Missing 9 = Inapplicable	If participant answered “yes” to either diabetes history question (or “borderline” in B4H1I), then participant has a history of diabetes. If there are discrepancies between the two, then code the individual as having a history of diabetes. Calculating Glucose_Points: If participant has … No history of diabetes (i.e., No for B4H1I and B4H1ID) and HbA1c < 5.7, then Glucose_Points = 100; No history of diabetes and HbA1c between 5.7 and 6.4, then Glucose_Points = 60; History of diabetes with HbA1c < 7.0, then Glucose_Points = 40; History of diabetes with HbA1c between 7.0 and 7.9, then Glucose_Points = 30; History of diabetes with HbA1c between 8.0 and 8.9, then Glucose_Points = 20; History of diabetes with HbA1c between 9.0 and 9.9, then Glucose_Points = 10; History of diabetes with HbA1c ≥10, then then Glucose_Points = 0.
Blood Pressure Subscore	Values are continuous and in mm Hg 997 = Don’t know (missing) 998 = Missing 999 = Inapplicable 1 = Yes 2 = No 7 = Don’t Know (missing) 8 = Refused 9 = Inappropriate	Note: Blood pressure numbers are systolic/diastolic Calculating BP_Points: If participant has a blood pressure of … <120/<80, then BP_Points = 100; 120–129/<80, then BP_Points = 75; 130–139 systolic OR 80–89 diastolic, then BP_Points = 50; 140–159 systolic OR 90–99 diastolic, then BP_Points = 25; ≥160 systolic OR ≥ 100 diastolic, then BP_Points = 0. If participant answers “Yes” to B1PA24C, then subtract 20 points from BP_Points score.

## References

[1] Lloyd-Jones D.M., Allen N.B., Anderson C.A.M., Black T., Brewer L.C., Foraker R.E., Grandner M.A., Lavretsky H., Perak A.M., Sharma G. (2022). Life’s Essential 8: Updating and enhancing the American Heart Association’s construct of cardiovascular health: A presidential advisory from the American Heart Association. Circulation.

[2] Lloyd-Jones D.M., Ning H., Labarthe D., Brewer L., Sharma G., Rosamond W., Foraker R.E., Black T., Grandner M.A., Allen N.B. (2022). Status of cardiovascular health in US adults and children using the American Heart Association’s new “Life’s Essential 8” metrics: Prevalence estimates from the National Health and Nutrition Examination Survey (NHANES), 2013 through 2018. Circulation.

[3] Li Y., Xia P.-F., Geng T.-T., Tu Z.-Z., Zhang Y.-B., Yu H.-C., Zhang J.-J., Guo K., Yang K., Liu G. (2023). Trends in self-reported adherence to healthy lifestyle behaviors among US adults, 1999 to March 2020. JAMA Netw. Open.

[4] Raffensperger S., Kuczmarski M.F., Hotchkiss L., Cotugna N., Evans M.K., Zongerman A.B. (2010). Effect of race and predictors of socioeconomic status on diet quality in the HANDLS Study sample. J. Natl. Med. Assoc..

[5] Whitaker K.M., Jacobs D.R., Kershaw K.N., Demmer R.T., Booth J.N., Carson A.P., Lewis C.E., Goff D.C., Lloyd-Jones D.M., Gordon-Larsen P. (2018). Racial disparities in cardiovascular health behaviors: The Coronary Artery Risk Development in Young Adults study. Am. J. Prev. Med..

[6] Ahn S., Lobo J.M., Logan J.G., Kang H., Kwon Y., Sohn M.-W. (2021). A scoping review of racial/ethnic disparities in sleep. Sleep Med..

[7] Sheehan C.M., Walsemann K.M., Alishire J.A. (2020). Race/ethnic differences in educational gradients in sleep duration and quality among U.S. adults. SSM Popul. Health.

[8] George K.M., Peterson R.L., Gilsanz P., Mungas D.M., Glymour M.M., Mayeda E.R., Whitmer R.A. (2020). Racial/ethnic differences in sleep quality among older adults: Kaiser Healthy Aging and Diverse Life Experiences (KHANDLE) study. Ethn. Dis..

[9] Cuevas A.G., Chen R., Slopen N., Thurber K.A., Wilson N., Economos C., Williams D.R. (2020). Assessing the role of health behaviors, socioeconomic status, and cumulative stress for racial/ethnic disparities in obesity. Obesity.

[10] Min J., Goodale H., Xue H., Brey R., Wang Y. (2021). Racial-ethnic disparities in obesity and biological, behavioral, and sociocultural influences in the United States: A systematic review. Adv. Nutr..

[11] Banas J., McDowell Cook A., Raygoza-Cortez K., Davila D., Irwin M.L., Ferrucci L.M., Humphries D.L. (2024). United States long-term trends in adult BMI (1959–2018): Unraveling the roots of the obesity epidemic. Int. J. Environ. Res. Public Health.

[12] Hardy S.T., Chen L., Cherrington A.L., Moise N., Jaeger B.C., Foti K., Sakhuja S., Wozniak G., Abdalla M., Muntner P. (2021). Racial and ethnic differences in blood pressure among US adults, 1999–2018. Hypertension.

[13] Aggarwal R., Chiu N., Wadhera R.K., Moran A.E., Raber I., Shen C., Yeh R.W., Kazi D.S. (2021). Racial/ethnic disparities in hypertension prevalence, awareness, treatment, and control in the United States, 2013 to 2018. Hypertension.

[14] Lee W., Lloyd J.T., Giuriceo K., Day T., Shrank W., Rajkumar R. (2020). Systematic review and meta-analysis of patient race/ethnicity, socioeconomics, and quality for adult type 2 diabetes. Health Serv. Res..

[15] Cavagnolli G., Pimentel A.L., Freitas P.A.C., Gross J.L., Camargo J.L. (2017). Effect of ethnicity on HbA1c levels in individuals without diabetes: Systematic review and meta-analysis. PLoS ONE.

[16] King B.A., Dube S.R., Tynan M.A. (2012). Current tobacco use among adults in the United States: Findings from the National Adult Tobacco Survey. Am. J. Public Health.

[17] Cornelius M.E., Loretan C.G., Jamal A., Lynn B.C.D., Mayer M., Alcantara I.C., Neff L. (2023). Tobacco product use among adults- United States, 2021. Morb. Mortal. Wkly. Rep..

[18] Howard G., Safford M.M., Moy C.S., Howard V.J., Kleindorfer D.O., Unverzagt F.W., Soliman E.Z., Flaherty M.L., McClure L.A., Lackland D.T. (2017). Racial differences in the incidence of cardiovascular risk factors in older Black and White adults. J. Am. Geriatr. Soc..

[19] Frank A.T.H., Zhao B., Jose P.O., Azar K.M.J., Fortmann S.P., Palaniappan L.P. (2014). Racial/ethnic differences in dyslipidemia patterns. Circulation.

[20] Tsao C.W., Aday A.W., Almarzooq Z.I., Alonso A., Beaton A.Z., Bettencourt M.S., Boehme A.K., Buxton A.E., Carson A.P., Commodore-Mensah Y. (2022). Heart disease and stroke statistics—2022 update: A report from the American Heart Association. Circulation.

[21] Mehta L.S., Velarde G.P., Lewey J., Sharma G., Bond R.M., Navas-Acien A., Fretts A.M., Magwood G.S., Yang E., Blumenthal R.S. (2023). Cardiovascular disease risk factors in women: The impact of race and ethnicity: A scientific statement from the American Heart Association. Circulation.

[22] Safford M.M., Gamboa C.M., Durant R.W., Brown T.M., Glasser S.P., Shikany J.M., Zweifler R.M., Howard G., Muntner P. (2015). Race-sex differences in the management of hyperlipidemia: The REasons for Geographic And Racial Differences in Stroke study. Am. J. Prev. Med..

[23] Michos E.D., Khan S.S. (2021). Further understanding of ideal cardiovascular health score metrics and cardiovascular disease. Expert Rev. Cardiovasc. Ther..

[24] Krefman A.E., Labarthe D., Greenland P., Pool L., Auayo L., Juonala M., Kähönen M., Lehtimäki T., Day R.S., Bazzano L. (2021). Influential periods in longitudinal clinical cardiovascular health scores. Am. J. Epidemiol..

[25] Lloyd-Jones D.M., Leip E.P., Larson M.G., D’Agostino R.B., Beiser A., Wilson P.W.F., Wolf P.A., Levy D. (2006). Prediction of lifetime risk for cardiovascular disease by risk factor burden at 50 years of age. Circulation.

[26] Dhingra R., Vasan R.S. (2012). Age as a cardiovascular risk factor. Med. Clin. N. Am..

[27] Ogunmoroti O., Osibogun O., Spatz E.S., Okunrintemi V., Mathews L., Ndumele C.E., Michos E.D. (2022). A systematic review of the bidirectional relationship between depressive symptoms and cardiovascular health. Prev. Med..

[28] Mathews L., Ogunmoroti O., Nasir K., Blumenthal R.S., Utuama O.A., Rouseff M., Das S., Veledar E., Feldman T., Agatston A. (2018). Psychological factors and their association with ideal cardiovascular health among women and men. J. Women’s Health.

[29] Hines A.L., Albert M.A., Blair J.P., Crews D.C., Cooper L.A., Long D.L., Carson A.P. (2023). Neighborhood factors, individual stressors, and cardiovascular health among Black and White adults in the US: The Reasons for Geographic and Racial Differences in Stroke (REGARDS) study. JAMA Open Netw..

[30] MacIntyre M.M., Zare M., Williams M.T. (2023). Anxiety-related disorders in the context of racism. Curr. Psychiatry Rep..

[31] Boehm J.K., Chen Y., Qureshi F., Soo J., Umukoro P., Hernandez R., Lloyd-Jones D., Kubzansky L.D. (2020). Positive emotions and favorable cardiovascular health: A 20-year longitudinal study. Prev. Med..

[32] Kubzansky L.D., Huffman J.C., Boehm J.K., Hernandez R., Kim E.S., Koga H.K., Feig E.H., Lloyd-Jones D.M., Seligman M.E.P., Labarthe D.R. (2018). Positive psychological well-being and cardiovascular disease: JACC Health Promotion series. J. Am. Coll. Cardiol..

[33] Levine G.N., Cohen B.E., Commodore-Mensah Y., Fleury J., Huffman J.C., Khalid U., Labarthe D.R., Lavretsky H., Michos E.D., Spatz E.S. (2021). Psychological health, well-being, and the mind-heart-body connection: A scientific statement from the American Heart Association. Circulation.

[34] Boehm J.K. (2021). Positive psychological well-being and cardiovascular disease: Exploring mechanistic and developmental pathways. Soc. Personal. Psychol. Compass.

[35] Boehm J.K., Peterson C., Mika K., Kubzansky L. (2011). A prospective study of positive psychological well-being and coronary heart disease. Health Psychol..

[36] Boehm J.K., Kubzansky L.D. (2012). The heart’s content: The association between positive psychological well-being and cardiovascular health. Psychol. Bull..

[37] Kubzansky L.D., Boehm J.K., Allen A.R., Vie L.L., Ho T.E., Trudel-Fitzgerald C., Koga H.K., Scheier L.M., Seligman M.E.P. (2020). Optimism and risk of incident hypertension: A target for primordial prevention. Epidemiol. Psychiatr. Sci..

[38] Boehm J.K., Soo J., Chen Y., Zevon E.S., Hernandez R., Lloyd-Jones D., Kubzansky L.D. (2017). Psychological well-being’s link with cardiovascular health in older adults. Am. J. Prev. Med..

[39] Cuevas A.G., Willams D.R., Albert M.A. (2017). Psychosocial factors and hypertension: A review of the literature. Cardiol. Clin..

[40] Trudel-Fitzgerald C., Gilsanz P., Mittleman M.A., Kubzansky L.D. (2015). Dysregulated blood pressure: Can regulating emotions help?. Curr. Hypertens. Rep..

[41] Love G.D., Seeman T.E., Weinstein M., Ryff C.D. (2010). Bioindicators in the MIDUS national study: Protocol, measures, sample, and comparative context. J. Aging Health.

[42] Sun J., Li Y., Zhao M., Yu X., Zhang C., Mangussen C.G., Xi B. (2023). Association of the American Heart Association’s new “Life’s Essential 8” with all-cause and cardiovascular disease-specific mortality: Prospective cohort study. BMC Med..

[43] Radloff L.S. (1977). The CES-D scales: A self-report depression scale for research in the general population. Appl. Psychol. Meas..

[44] Patrick C.J., Curtin J.J., Tellegen A. (2002). Development and validation of a brief form of the Multidimensional Personality Questionnaire. Psychol. Assess..

[45] Scheier M.F., Carver C.S. (1985). Optimism, coping and health: Assessment and implications of generalized outcome expectancies. Health Psychol..

[46] Cohen S., Kamarck T., Mermelstein R. (1983). A global measure of perceived stress. J. Health Soc. Behav..

[47] Spielberger C.D., Gorsuch R.L., Lushene R., Vagg P.R., Jacobs G.A. (1983). Manual for the State-Trait Anxiety Inventory.

[48] Ryff C.D. (1989). Happiness is everything, or is it? Explorations on the meaning of psychological well-being. J. Personal. Soc. Psychol..

[49] Shapiro S.L., Carlson L.E. (2009). The Art and Science of Mindfulness: Integrating Mindfulness into Psychology and the Helping Professions.

[50] Emmons R.A., McCullough M.E. (2003). Counting blessings versus burdens: An experimental investigation of gratitude and subjective well-being in daily life. J. Personal. Soc. Psychol..

[51] Baer R.A. (2003). Mindfulness training as a clinical intervention: A conceptual and empirical review. Clin. Psychol. Sci. Pract..

[52] Adler N.E., Epel E.S., Castellazzo G., Ickovics J.R. (2000). Relationship of subjective and objective social status with psychological and physiological functioning: Preliminary data in healthy White women. Health Psychol..

[53] Shetty N.S., Parcha V., Patel N., Yadav I., Basetty C., Li C., Pandey A., Kalra R., Li P., Arora G. (2023). AHA Life’s Essential 8 and ideal cardiovascular health among young adults. Am. J. Prev. Cardiol..

[54] Tsenkova V.K., Love G.D., Singer B.H., Ryff C.D. (2008). Coping and positive affect predict longitudinal change in glycosylated hemoglobin. Health Psychol..

[55] Oreskovic N.M., Goodman E. (2013). Association of optimism with cardiometabolic risk in adolescents. J. Adolesc. Health.

[56] Patterson S.L., Marcus M., Goetz M., Vaccarino V., Gooding H.C. (2022). Depression and anxiety are associated with cardiovascular health in young adults. J. Am. Heart Assoc..

[57] Geronimus A.T. (1992). The weathering hypothesis and the health of African American women and infants: Evidence and speculations. Ethn. Dis..

[58] Simons R.L., Lei M.-K., Klopack E., Zhang Y., Gibbons F.X., Beach S.R.H. (2020). Racial discrimination, inflammation, and chronic illness among African American women at midlife: Support for the weathering perspective. J. Racial Ethn. Health Disparities.

[59] Schwalm F.D., Zandavalli F.B., Dias de Castro Filho E., Lucchetti G. (2021). Is there a relationship between spirituality/religiosity and resilience? A systematic review and meta-analysis of observational studies. J. Health Psychol..

[60] Stamps D.L., Mandell L., Lucas R. (2021). Relational maintenance, collectivism, and coping strategies among Black populations during COVID-19. J. Soc. Pers. Relatsh..

[61] Bey G.S., Jesdale B., Forrester S., Person S.D., Kiefe C. (2019). Intersectional effects of racial and gender discrimination on cardiovascular health vary among Black and White women and men in the CARDIA study. SSM-Popul. Health.

[62] Brown L.L., Mitchell U.A., Ailshire J.A. (2020). Disentangling the stress process: Race/ethnic differences in the exposure and appraisal of chronic stressors among older adults. J. Gerontol. Ser. B Psychol. Sci. Soc. Sci..

[63] Lee C., Park S., Boylan J.M. (2021). Cardiovascular health at the intersection of race and gender: Identifying life-course processes to reduce health disparities. J. Gerontol. Ser. B Psychol. Sci. Soc. Sci..

[64] Magee W., Elliott M.R., Sinkewicz M., Finlay J., Clarke P. (2022). Who looks on the bright side? Optimistic and pessimistic perceptual-response reflexes over American adulthood. Adv. Life Course Res..

[65] Gou Y., Cheng S., Kang M., Zhou R., Liu C., Hui J., Liu Y., Wang B., Shi P., Zhang F. (2025). Association of allostatic load with depression, anxiety, and suicide: A prospective cohort study. Biol. Psychiatry.

[66] Carbone J.T. (2020). Allostatic load and mental health: A latent class analysis of physiological dysregulation. Stress.

[67] Guidi J., Lucente M., Sonino N., Fava G.A. (2021). Allostatic load and its impact on health: A systematic review. Psychother. Psychosom..

[68] Mauss D., Jarczok M.N. (2021). The streamlined allostatic load index is associated with perceived stress in life- Findings from the MIDUS study. Stress.

[69] Huffman J.C., Feig E.H., Millstein R.A., Freedman M., Healy B.C., Chung W.-J., Amonoo H.L., Malloy L., Slawsby E., Januzzi J.L. (2019). Usefulness of a positive psychology-motivational interviewing intervention to promote positive affect and physical activity after an acute coronary syndrome. Am. J. Cardiol..

[70] Celano C.M., Albanese A.M., Millstein R.A., Mastromauro C.A., Chung W.-J., Campbell K.A., Legler S.R., Park E.R., Healy B.C., Collins L.M. (2019). Optimizing a positive psychology intervention to promote health behaviors following an acute coronary syndrome: The Positive Emotions after Acute Coronary Events-III (PEACE-III) randomized factorial trial. Psychosom. Med..

[71] Huffman J.C., Golden J., Massey C.N., Feig E.H., Chung W.-J., Millstein R.A., Brown L., Gianangelo T., Healy B.C., Wexler D.J. (2021). A positive psychology-motivational interviewing program to promote physical activity in type 2 diabetes: The BEHOLD-16 pilot randomized trial. Gen. Hosp. Psychiatry.

[72] Boutin-Foster C., Offidani E., Kanna B., Ogedegbe G., Revenell J., Scott E., Rodriguez A., Ramos R., Michelen W., Gerber L.M. (2016). Results from the trial using motivational interviewing, positive affect,, and self-affirmation in African Americans with Hypertension (TRIUMPH). Ethincity Dis..

[73] Tönis K.J.M., Kraiss J.T., Linssen G.C.M., Bohlmeijer E.T. (2023). The effects of positive psychology interventions on well-being and distress in patients with cardiovascular diseases: A systematic review and meta-analysis. J. Psychosom. Res..

[74] Park J.W., Mealy R., Saldanha I.J., Loucks E.B., Needham B.L., Sims M., Fava J.L., Dulin A.J., Howe C.J. (2021). Multilevel resilience resources and cardiovascular disease in the United States: A systematic review and meta-analysis. Health Psychol..

[75] Boehm J.K., Chen Y., Koga H., Mathur M.B., Vie L.L., Kubzansky L.D. (2018). Is optimism associated with healthier cardiovascular-related behavior? Meta-analyses of 3 health behaviors. Circ. Res..

[76] Ait-Hadad W., Bénard M., Shankland R., Kesse-Guyot E., Robert M., Touvier M., Hercberg S., Buscail C., Péneau S. (2020). Optimism is associated with diet quality, food group consumption and snacking behavior in a general population. Nutr. J..

[77] Mulkana S.S., Hailey B.J. (2001). The role of optimism in health-enhancing behavior. Am. J. Health Behav..

[78] Rose G. (2001). Sick individuals and sick populations. Int. J. Epidemiol..

[79] Singh S.S., Stranges S., Wilk P., Tang A.S.L., Frisbee S.J. (2023). Influence of the social environment on ideal cardiovascular health. J. Am. Heart Assoc..

[80] Gebreab S.Y., Davis S.K., Symanzik J., Mensah G.A., Gibbons G.H., Diez-Roux A.V. (2015). Geographic variations in cardiovascular health in the United States: Contributions of state- and individual-levle factors. J. Am. Heart Assoc..

[81] Foraker R.E., Bush C., Greiner M.A., Sims M., Henderson K., Smith S., Bidulescu A., Shoben A.B., Hardy N.C., O’Brien E. (2019). Distribution of cardiovascular health by individual- and neighborhood-level socioeconomic status: Findings from the Jackson Heart Study. Glob. Heart.

[82] Javed Z., Maqsood M.H., Yahya T., Amin Z., Acquah I., Valero-Elizondo J., Andrieni J., Dubey P., Jackson R.K., Daffin M.A. (2022). Race, racism, and cardiovascular health: Applying a social determinants of health framework to racial/ethnic disparities in cardiovascular disease. Circ. Cardiovasc. Qual. Outcomes.

[83] Agurs-Collins T., Persky S., Paskett E.D., Barkin S.L., Meissner H.I., Nansel T.R., Arteaga S.S., Zhang X., Das R., Farhat T. (2019). Designing and assessing multilevel interventions to improve minority health and reduce health disparities. Am. J. Public Health.

[84] Sims M., Glover L.M., Norwood A.F., Jordan C., Min Y.-I., Brewer L.C., Kubzansky L.D. (2019). Optimism and cardiovascular health among African Americans in the Jackson Heart Study. Prev. Med..

